# miR-17-5p drives G2/M-phase accumulation by directly targeting CCNG2 and is related to recurrence of head and neck squamous cell carcinoma

**DOI:** 10.1186/s12885-021-08812-6

**Published:** 2021-10-02

**Authors:** Qiang Huang, Yu-Jie Shen, Chi-Yao Hsueh, Yang Guo, Yi-Fan Zhang, Jiao-Yu Li, Liang Zhou

**Affiliations:** 1 Department of Otorhinolaryngology, Eye & ENT Hospital, Fudan University, Shanghai, 200031 China; 2 Department of Pediatric, Xinhua Hospital, Shanghai Jiaotong University School of Medicine, Shanghai, 200092 China

**Keywords:** miR-17-5p, HNSCC, Cell cycle, CCNG2, Recurrence

## Abstract

**Background:**

The human miR-17-92 polycistron is the first reported and most well-studied onco-miRNA with a cluster of seven miRNAs. miR-17-5p, a member of the miR-17-92 family, plays an important role in tumor cell proliferation, apoptosis, migration and invasion. However, few studies have shown the role of miR-17-5p in the cell cycle of head and neck squamous cell carcinoma (HNSCC).

**Methods:**

RT-qPCR was used to detect miR-17-5p expression levels in 64 HNSCC tissues and 5 cell lines. The relationship between the expression of miR-17-5p in the tissues and the clinical characteristics of the patients was analyzed. HNSCC cells were transfected with an miR-17-5p mimic or inhibitor to evaluate cell cycle distribution by flow cytometry. Cell cycle distribution of cells transfected with target gene was evaluated using flow cytometry. Dual-luciferase reporter assay was used to detect the regulatory effect of miR-17-5p on target gene expression.

**Results:**

In the present study, we found that miR-17-5p expression in HNSCC tissues and cell lines was remarkably increased, and miR-17-5p is related to recurrence in HNSCC patients. Silencing miR-17-5p blocked HNSCC cells in G2/M phase, whereas its overexpression propelled cell cycle progression. More importantly, we verified that miR-17-5p negatively regulated CCNG2 mRNA and protein expression by directly targeting its 3’UTR.

**Conclusion:**

These findings suggest that miR-17-5p might act as a tumor promoter and prognostic factor for recurrence in HNSCC patients.

## Introduction

Head and neck squamous cell carcinoma (HNSCC) was the seventh most common cancer worldwide in 2018 (890,000 new cases and 450,000 deaths), accounting for 3% of all cancers and just over 1.5% of all cancer deaths in the United States [1]. Despite advances in diagnosis and treatment, recurrent, or metastatic disease (or both) develops in more than 65% of patients with head and neck cancer [2]. Therefore, a better understanding of the molecular mechanisms of HNSCC is an urgent matter.

The cell cycle is a highly organized and systematically controlled process. Dysregulation of cell cycle control, such as unscheduled proliferation, is regarded as one of the key drivers of genomic and chromosomal instability that facilitates tumorigenesis [3, 4]. MicroRNAs (miRNAs) are vital post-transcriptional modulators that directly target mRNA at the 3′-untranslated region (UTR) for transcriptional repression or degradation [5]. miRNAs are involved in the progression of the cell cycle of cancer cells [6, 7]. The human miR-17-92 polycistron is the first reported and most well-studied onco-miRNA with a cluster of seven miRNAs, including miR-17-5p, derived from the c-myc-regulated C13orf25 locus at chromosome 13q31.3 [8]. miR-17-5p has been implicated in cancer development, including proliferation, apoptosis, migration, and invasion. Wang et al. found that dysregulation of miR-17-5p/PIK3R1 axis participated in laryngeal squamous cell carcinoma (LSCC) cell proliferation and apoptosis by inhibiting the activation of the PI3K/AKT signaling pathway [5]. Zhu et al. demonstrated that miR-17-5p enhanced cancer cell proliferation by altering cell cycle profiles via the disruption of RBL2/E2F4-repressing complexes in pancreatic cancer [9]. In our previous study, we presented comprehensive profiling of miRNAs in HNSCC. Several miRNAs were validated, including upregulation of miR-17, miR-21, miR-93, miR-205 and miR-708 and downregulation of miR-125b and miR-145 [10]. However, few studies have shown the role of miR-17-5p in the cell cycle of HNSCC.

The cell cycle is controlled by cyclin-dependent protein kinase (CDK), and its activity is regulated by cyclin and CDK inhibitors [11]. The eight cyclins reported in mammals, cyclins A to H, share a conservative amino acid sequence of about 90 residues, called the cyclin box. The amino acid sequence of cyclin G is very conserved in mammals. The nucleotide sequences of cyclin G1 and cyclin G2 are 53% identical [12]. Unlike cyclin G1, cyclin G2 (CCNG2) contains a C-terminal protein epitope signature tag (PEST) protein destabilizing motif, indicating that cyclin G2 expression is strictly regulated during the cell cycle. Although CCNG2 has been found to be connected with cancer stemness and chemoresistance of HNSCC cells [13], the role of CCNG2 during cell cycle process of HNSCC has not been completely understood.

This study aims to clarify the role of miR-17-5p in the cell cycle process of HNSCC and the correlation with clinical characteristics of patients with HNSCC, and to study the target genes that miR-17-5p may regulate by bioinformatics methods and dual-luciferase reporter assay exploring the mechanism of miR-17-5p related cell cycle changes in HNSCC.

## Methods and material

### Patient information and ethics approval

Formalin-fixed and paraffin-embedded HNSCC tissues (mainly laryngeal cancer and hypopharyngeal cancer) were obtained from 64 patients diagnosed with HNSCC pathologically after surgery from April 2010 to August 2010 from the Department of Otorhinolaryngology, Eye & ENT Hospital of Fudan University. The inclusion criteria were as follows: (a) presence of a signed informed consent form obtained before the operation and (b) confirmation of HNSCC by experienced pathologists and classification of tumor stage according to the 8th edition of the AJCC cancer staging manual with complete clinical, imaging, laboratory, and pathological data. The exclusion criteria were as follows: (a) histopathological confirmation of multiple types of primary HNSCC, (b) preoperative treatment with approaches such as radiotherapy or chemotherapy, or (c) infectious disease or autoimmune disease.

In parallel, tissues obtained from 18 patients diagnosed with vocal cord polyps were used as non-tumor control. Relevant clinicopathological and prognostic information was collected. All participants provided written informed consent. The protocols were authorized by the Clinical Research Ethics Committee of the Eye & ENT Hospital of Fudan University (NO.KJ2008–01).

### Cell culture

The HNSCC cell line AMC-HN8, which was established by Kim et al. [14] in 1997 from patients with head and neck cancer, was maintained in our laboratory. Tu686 was obtained from Central South University (Hunan, China). FaDu and Detroit562 were obtained from the Cell Bank of the Shanghai Institute of Cells, Chinese Academy of Science (Shanghai, China). HNSCC cell lines were cultured in DMEM (Gibco, Grand Island, NY), except for AMC-HN8, which was cultured in RPMI-1640 (HyClone, Logan, UT). Growth medium contained 1% penicillin-streptomycin (Genom Biotechnology, China) and 10% fetal bovine serum (FBS; Gibco, Grand Island, NY). Cells were incubated with 5% CO2 at 37 °C. HuLa-PC, a cell line derived from posterior commissure of the larynx, was obtained from ATCC (Gaithersburg, Maryland) and cultured in Dermal Cell Basal Medium (ATCC® PCS-200-030™) supplied with Keratinocyte Growth Kit (ATCC® PCS-200-040™) [15].

### RT-qPCR

Total RNA was isolated from tissues with an RNeasy FFPE Kit (QIAGEN, Germany) and cell lines with TRIzol reagent (Invitrogen, Thermo Fisher Scientific) and then reversed-transcribed using an miScript II RT Kit (QIAGEN). qRT-PCR was conducted using the miScript SYBR Green PCR Kit for miRNA using U6 as an internal control and QuantiNova SYBR® Green PCR Kit for mRNA (both from QIAGEN) with the ABI 7500 Real-Time PCR System (Life Technologies, Shanghai, China). The primers were synthesized by Sangon Biotech (Shanghai). The sequences of all primers used are listed in Table 1.

**Table 1:** Sequences of primers

Primer	Forward	Reverse
miR-17-5p	CCAAAGTGCTTACAGTGCAGGTA	N/A
Cyclin A2	GGATGGTAGTTTTGAGTCACCAC	CACGAGGATAGCTCTCATACTGT
Cyclin B1	AATAAGGCGAAGATCAACATGGC	TTTGTTACCAATGTCCCCAAGAG
Cyclin D1	GCTGCGAAGTGGAAACCATC	CCTCCTTCTGCACACATTTGAA
Cyclin E2	TCAAGACGAAGTAGCCGTTTAC	TGACATCCTGGGTAGTTTTCCTC
Cyclin G2	TCTCGGGTTGTTGAACGTCTA	GTAGCCTCAATCAAACTCAGCC
CDK1	AAACTACAGGTCAAGTGGTAGCC	TCCTGCATAAGCACATCCTGA
CDK2	GTACCTCCCCTGGATGAAGAT	CGAAATCCGCTTGTTAGGGTC
CDK4	TCAGCACAGTTCGTGAGGTG	GTCCATCAGCCGGACAACAT
CDK6	TCTTCATTCACACCGAGTAGTGC	TGAGGTTAGAGCCATCTGGAAA
GAPDH	TGTAGTTGAGGTCAATGAAGGG	ACATCGCTCAGACACCATG

### Plasmid, siRNA and miRNA transfection

Hsa-miR-17-5p mimic, 5′-CAAAGUGCUUACAGUGCAGGUAG-3′ (sense) and 5′-ACCUGCACUGUAAGCACUUUGUU-3′ (antisense), and inhibitor, 5′-CUACCUGCACUGUAAGCACUUUG-3′ (sense), were designed and synthesized by Sangon Biotech (Shanghai). The CCNG2 overexpression plasmid (NM_004354.3) and empty plasmid were obtained from Genomeditech (Shanghai, China). The transfection was performed with Lipofectamine 2000 (Invitrogen, Carlsbad, CA) according to the manufacturer’s instructions.

### Cell cycle assay

The effects of miR-17-5p and CCNG2 on the progress of the cell cycle were investigated using the propidium iodide technique. Briefly, after transfecting with miRNA, siRNA, or plasmid for 24 h, the HNSCC cells were starved for further 24 h in serum-free culture medium to synchronize. Cells were then fixed in 75% pre-cooling ethanol overnight at − 20 °C, washed twice with PBS, and then incubated with 500 μL PI/RNase staining buffer (BD Biosciences) for 15 min in dark. Cells were then tested using flow cytometry (SP8 MoFlo XDP, Beckman Coulter), and the cell cycle distribution was investigated with FlowJo Software (FlowJo LLC).

### Western blotting

Protein concentrations were detected with BCA (Beyotime, China). Rabbit anti-CCNG2 antibody (Sigma-Aldrich, Cat. #HPA034684, 1:1000) in 5% BSA were used. Refer to our previous articles for the rest of the steps [16, 17].

### Dual-luciferase reporter assay

The binding sites between miR-17-5p and CCNG2 were predicted by Targetscan. 293 T cells were co-transfected with CCNG2-wild-type (WT) 3′ untranslated region (UTR) luciferase reporter/CCNG2-mutant-type (MU) 3′UTR luciferase reporter and miR-17-5p mimic/mimic NC. Luciferase activities were measured 48 h after transfection.

### Database information

The ENCORI Pan-Cancer Analysis Platform (http://starbase.sysu.edu.cn/index.php) was used to perform a differential expression analysis of genes.

### Statistical analysis

Statistical analysis was performed using Student’s t-test or one-way analysis of variance (ANOVA), except if specified otherwise in the figure legend. Results are expressed as the means ± standard deviation (SD). A *p* value was considered significant as the following: * *p* < 0.05; ** *p* < 0.01; *** *p* < 0.001. Results that were not statistically significant are labeled as ns. Data were plotted and analyzed using GraphPad Prism 8.

## Results

### miR-17-5p is upregulated in HNSCC tissues and cell lines and is related to HNSCC recurrence

**Fig. 1:**
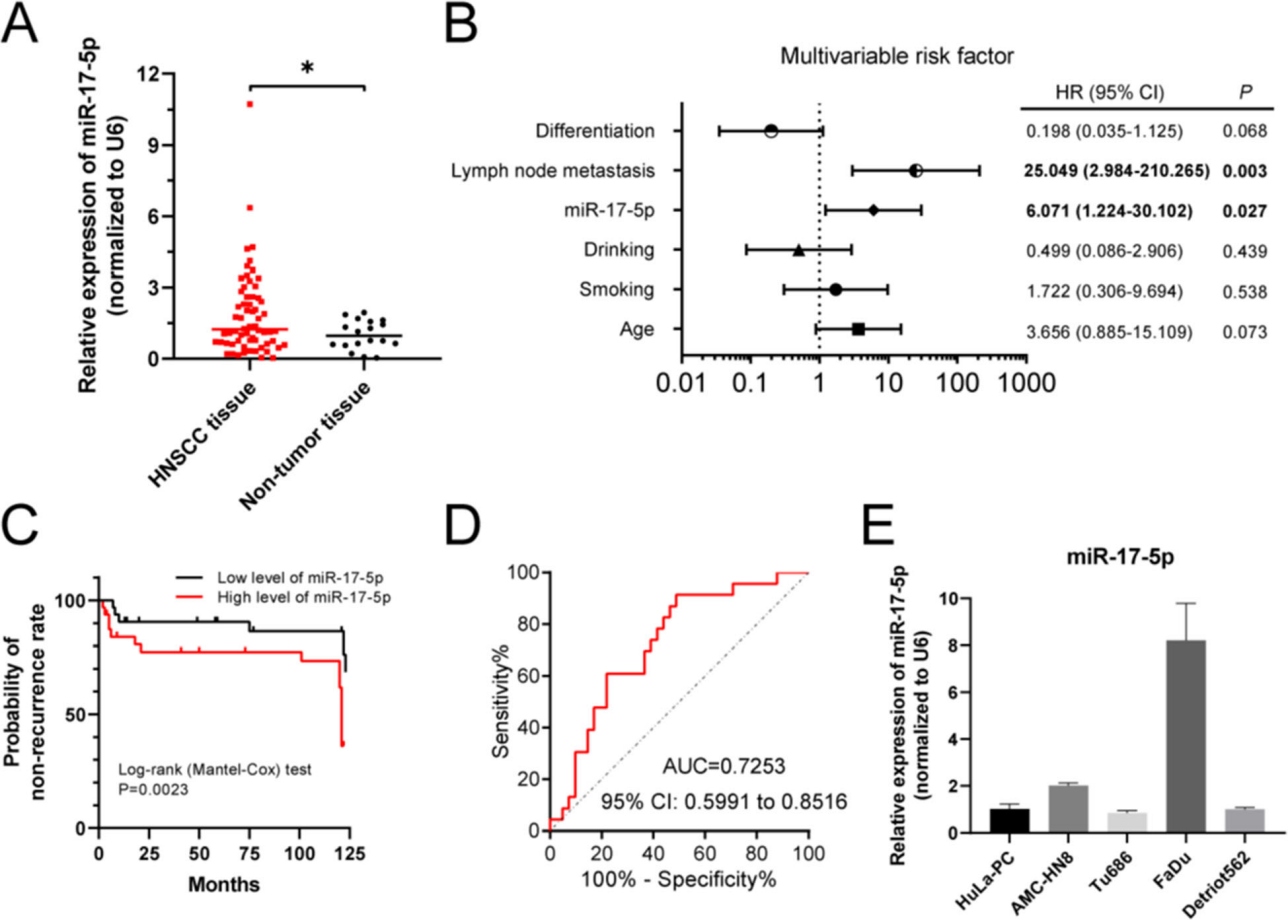
miR-17-5p is increased in HNSCC tissues and cell lines. **(A)** qRT-PCR was used to detected miR-17-5p expression level in HNSCC tissues (*n* = 64) and non-tumor tissues (*n* = 18). Normalized to U6. *p < 0.01 vs. normal tissues. **(B)** Multivariable risk factor and 95% confidence interval for HNSCC. Lymph node metastasis (HR = 25.049) and miR-17-5p (HR = 6.071) were independent risk factors for HNSCC patients (*P* < 0.05). **(C)** Log-rank (Mantel-Cox) test for the non-recurrence rate of HNSCC patients with low or high level of miR-17-5p. *P* = 0.0023. **(D)** The diagnostic potential of miR-17-5p in HNSCC patients. The area under the ROC curve was 0.7253 (95% CI: 0.5991 to 0.8516; *p* < 0.0001). **(E)** qRT-PCR was used to detect miR-17-5p expression level in four HNSCC cell lines (AMC-HN8, Tu686, FaDu, and Detriot562) compared with human posterior commissure of the larynx cell line HuLa-PC

To investigate the role of miR-17-5p in HNSCC development, we first ascertained the level of miR-17-5p in HNSCC tissues. qRT-PCR result showed that miR-17-5p level was significantly upregulated in HNSCC tissues (*n* = 64) compared with that in vocal cord polys tissues (*n* = 18) (Fig. 1A). Univariate analysis revealed that miR-17-5p levels in HNSCC tissues of patients with recurrence (*n* = 23) were significantly higher than those of patients without recurrence (*n* = 41; *p* = 0.019; Table 2). Multivariate analysis showed that lymph node metastasis (*p* = 0.003) and miR-17-5p (*p* = 0.027) were independent risk factors for HNSCC (Fig. 1B).

**Table 2:** Clinicopathologic characteristics of HNSCC patients

Features	All cases	miR-17-5p		*p* value
		Low (*n* = 32)	High (*n =* 32)	
Age (years)				0.617
	≤60	17	15	
	> 60	15	17	
Gender				0.551
	Male	31	30	
	Female	1	2	
Smoking				0.309
	No	15	11	
	Yes	17	21	
Drinking				0.448
	No	17	20	
	Yes	15	12	
Hypertension				0.442
	No	21	18	
	Yes	11	14	
Diabetes				0.719
	No	28	27	
	Yes	4	5	
Lymph node metastasis				0.768
	Negative	25	24	
	Positive	7	8	
TNM stage				0.076
	I + II	17	10	
	III + IV	15	22	
Differentiation				0.062
	Well+ well-moderately	14	7	
	Moderately+ Moderately-poorly	18	25	
Recurrence				**0.019**
	No	25	16	
	Yes	7	16	
Death				0.095
	No	26	20	
	Yes	6	12	

Importantly, we found that HNSCC patients with higher levels of miR-17-5p had a higher likelihood of recurrence compared with patients who had a lower level of miR-17-5p (*p* = 0.0023; Fig. 1C). The AUC for miR-17-5p distinguishing HNSCC patients with recurrence from patients without recurrence was 0.7253 (95% CI: 0.5991 to 0.8516; *p* < 0.0029; Fig. 1D), suggesting that miR-17-5p is related to recurrence in HNSCC patients (sensitivity: 60.87%, specificity: 78.05%).

Moreover, we examined the miR-17-5p expression levels in disparate HNSCC cell lines (AMC-HN8, Tu686, FaDu, and Detriot562) compared with that in the human posterior commissure of the larynx cell line HuLa-PC. Levels of miR-17-5p were higher in AMC-HN8 and FaDu than in HuLa-PC but did not increase in Tu686 and Detriot562 (Fig. 1E). These results suggest that the miR-17-5p is upregulated in HNSCC and may be correlated with HNSCC recurrence.

### miR-17-5p promotes cell cycle progress in HNSCC in vitro

**Fig. 2:**
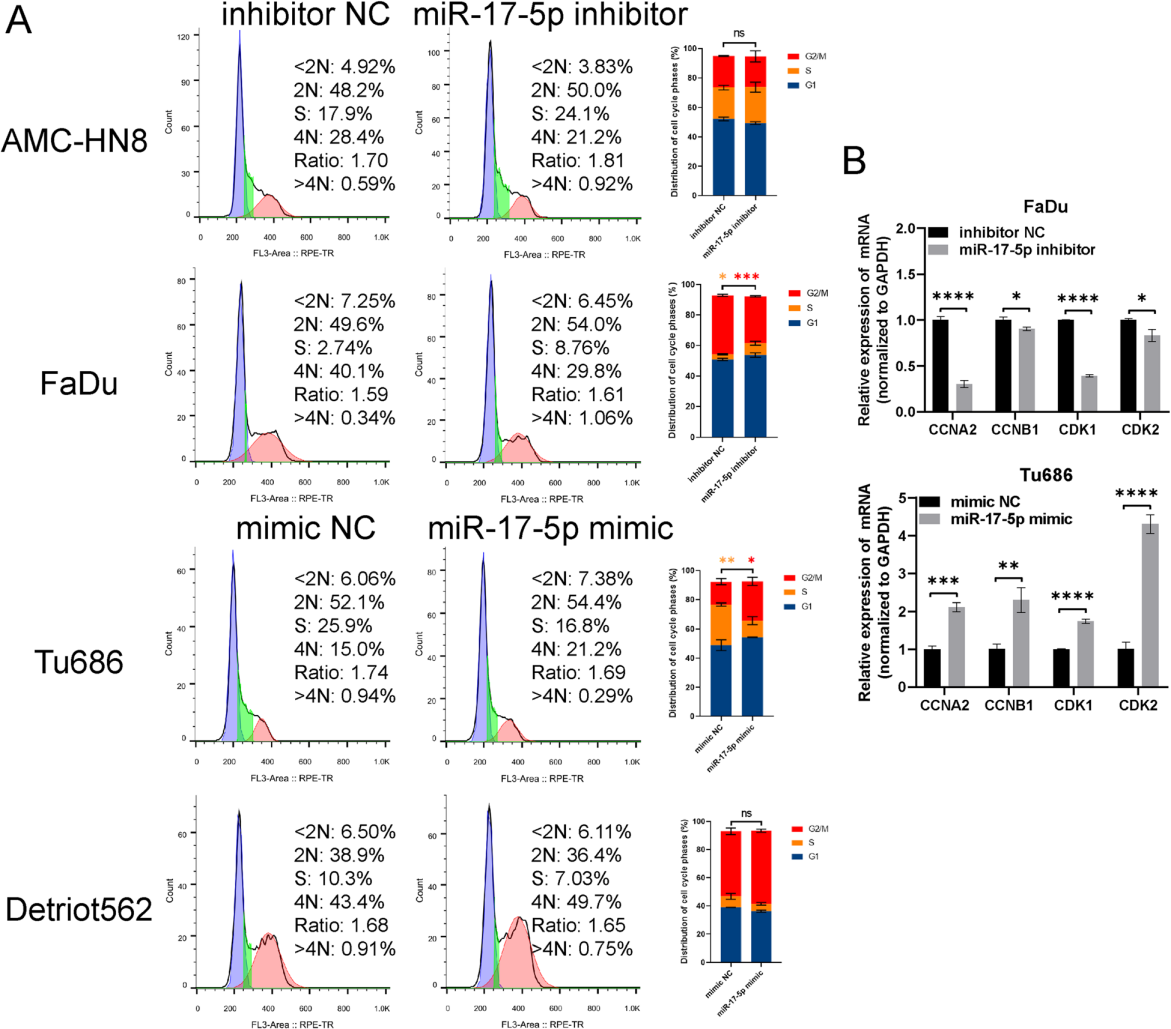
miR-17-5p promotes cell cycle progress in HNSCC in vitro. **(A)** AMC-HN8 cells and FaDu cells were transfected with miR-17-5p inhibitor or inhibitor NC. Tu686 cells and Detriot562 cells were transfected with miR-17-5p mimic or mimic negative control (NC). Cell cycle phase distribution was detected by flow cytometry. Data shown are the mean ± standard deviation of three independent experiments. *p < 0.05, **p < 0.01, ***p < 0.001 vs. inhibitor NC or mimic NC. **(B)** Cell cycle-related genes were detected by qRT-PCR. *p < 0.05, **p < 0.01, ***p < 0.001 vs. inhibitor NC or mimic NC

Previous studies have confirmed that miR-17-5p plays a role in promoting cell cycle progression as an onco-miRNA associated with a variety of cancers [9, 18]. Consequently, we aimed to investigate whether miR-17-5p is involved in HNSCC cell cycle. Cell cycle phase distribution was detected by flow cytometry. The inhibition of miR-17-5p resulted in an increase in cells at the S phase (from 2.74 to 8.76%) and a decrease in cells at G2/M phase (from 40.1 to 29.8%) in FaDu cells, not in AMC-HN8. By contrast, the overexpression of miR-17-5p in Tu686 cells, not in Detriot562, led to a decrease in cells at the S phase (from 25.9 to 16.8%) and an increase in cells at G2/M phase (from 15 to 21.2%, Fig. 2A). We also detected the expression levels of cell cycle-related genes in the two HNSCC cells. We found that CCNA2, CCNB1 and CDK1/2, which regulate cell cycle progress, were decreased in miR-17-5p inhibitor-transfected FaDu cells but increased in miR-17-5p mimic-transfected Tu686 cells (Fig. 2B). Therefore, these results suggest that miR-17-5p might play a role in promoting cell cycle progress in HNSCC in vitro.

### CCNG2 is a direct target gene of miR-17-5p

**Fig. 3:**
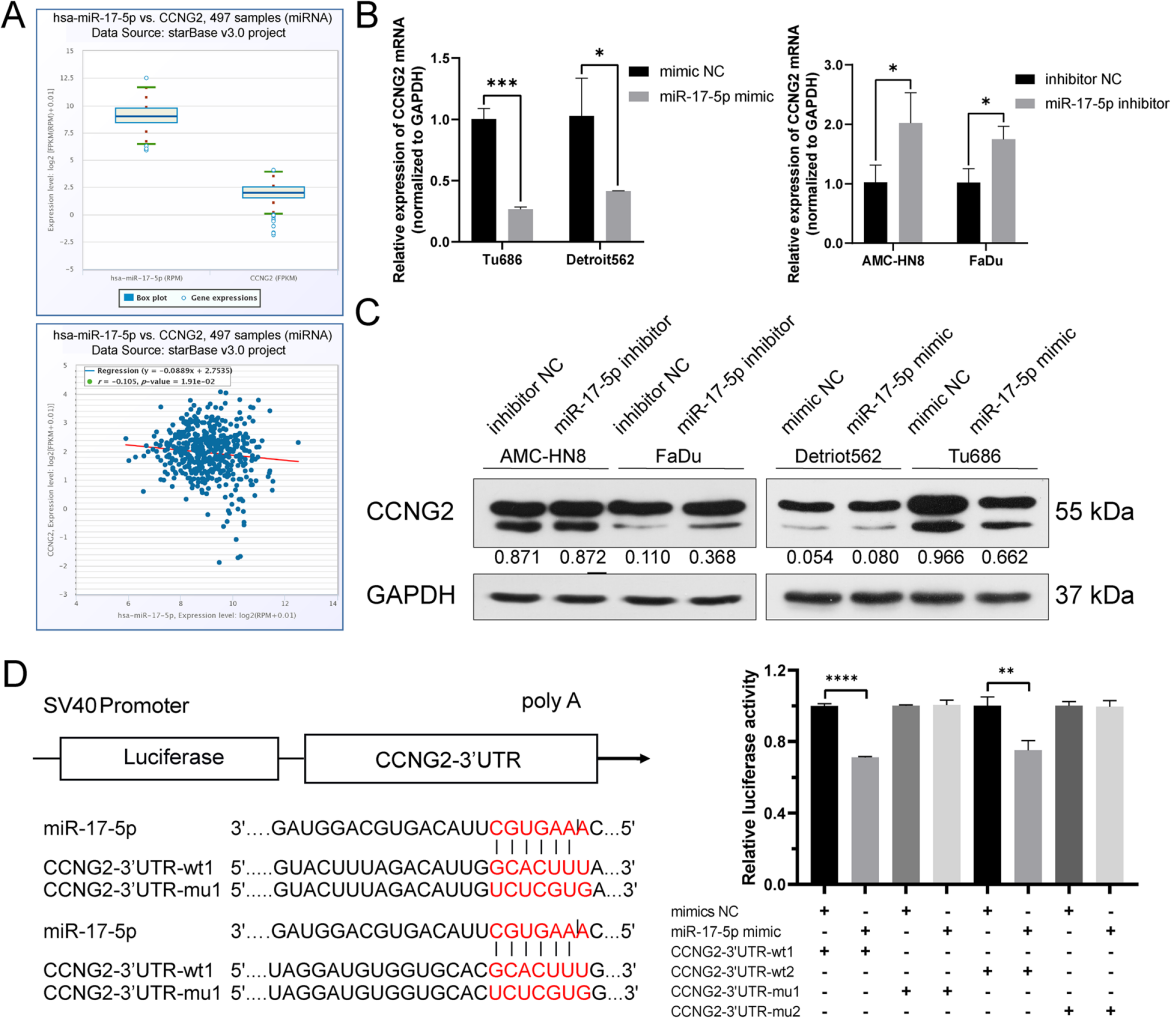
CCNG2 is a direct target gene of miR-17-5p. **(A)** Bioinformatics results for miR-17-5p and CCNG2 expression level (log2[FPKM (RPM) + 0.01]) in 497 samples of HNSCC (up panel). The co-expression relation of miR-17-5p and CCNG2 expression level in 497 samples of HNSCC (down panel). r = − 0.105, *p* = 0.0191. **(B)** qRT-PCR and **(C)** western blot were used to detect the CCNG2 mRNA and protein level after miR-17-5p mimic or inhibitor transfection. *p < 0.05 vs. mimic NC or inhibitor NC. Gray value analysis is shown below the band (normalized to GAPDH). **(D)** Potential binding site of miR-17-5p at the 3’UTR of CCNG2 mRNA. **(E)** 293 T cells were co-transfected with miR-17-5p mimic or mimic NC and luciferase reporter plasmid containing wild-type or mutated miR-17-5p-binding site at CCNG2 3’UTR. Dual-luciferase reporter assay was used to detect luciferase activity. **p < 0.01, ****p < 0.0001 vs. mimic NC

In order to study the mechanism by which miR-17-5p drives HNSCC cell cycle changes, we examined the cell cycle regulators possibly targeted by miR-17-5p. Using the bioinformatics tool, we identified CCNG2 mRNA as one of the putative targets of miR-17-5p due to its involvement in the inhibition of tumor proliferation and cell cycle [7, 19]. We found that miR-17-5p expression level was higher than that of CCNG2 in 497 HNSCC samples, and there was a significant negative correlation between miR-17-5p and CCNG2 (r = − 0.105, *p* = 0.0191; Fig. 3A). In AMC-HN8 and FaDu cells, the inhibition of miR-17-5p almost doubled CCNG2 mRNA levels compared with cells transfected with the negative control, and overexpression of miR-17-5p in Tu686 and Detriot562 led to a decrease in CCNG2 mRNA of approximately 60–75% (Fig. 3B). Similarly, the inhibition of miR-17-5p increased the CCNG2 protein level (only in FaDu cells), which was reversed following the overexpression of miR-17-5p (only in Tu686 cells) (Fig. 3C).

To determine whether CCNG2 is a direct target gene of miR-17-5p, we performed a dual-luciferase reporter assay. The binding sites for miR-17-5p in the 3’UTR of CCNG2 are illustrated in Fig. 3D. CCNG2–3’UTR wt1/2 or CCNG2–3’UTR-mu1/2 was co-transfected with miR-17-5p mimic or mimic NC into 293 T cells. Fluorescence intensity was decreased by 25–29% in cells co-transfected with miR-17-5p mimic and CCNG2–3’UTR-wt1/2. However, neither the miR-17-5p mimic nor mimic NC affected the fluorescence intensity of cells transfected with CCNG2–3’UTR-mu1/2 (Fig. 3E). These findings indicate that miR-17-5p directly targets CCNG2, which may drive the alteration of the HNSCC cell cycle.

### CCNG2 inhibits cell cycle progress in HNSCC in vitro

**Fig. 4:**
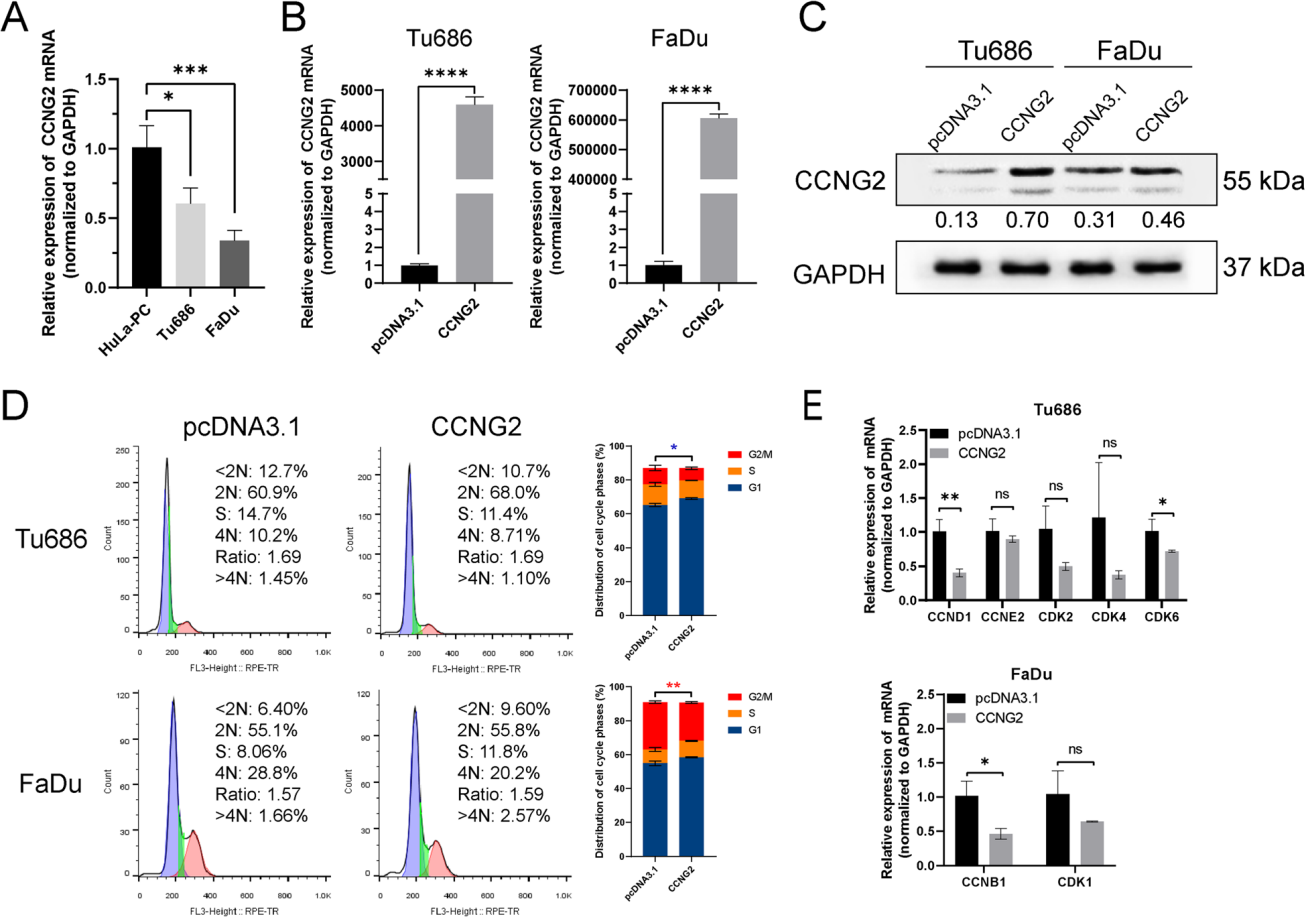
CCNG2 inhibits cell cycle progress in HNSCC in vitro. **(A)** qRT-PCR was used to detect the intracellular expression level in HNSCC cell lines Tu686 and FaDu compared with HuLa-PC cells. *p < 0.05, ***p < 0.001 vs. HuLa-PC cells. **(B)** qRT-PCR and **(C)** western blot were used to detect the CCNG2 mRNA and protein levels after plasmid transfection. ****p < 0.0001 vs. pcDNA3.1 group. Gray value analysis is shown below the band (normalized to GAPDH). **(D)** Tu686 cells and FaDu cells were transfected with CCNG2 plasmid or pcDNA3.1. Cell cycle phase distribution was detected by flow cytometry. Data shown are the mean ± standard deviation of three independent experiments. *p < 0.05, **p < 0.01 vs. pcDNA3.1 group. **(E)** Cell cycle-related genes were detected by qRT-PCR. *p < 0.05, **p < 0.01 vs. pcDNA3.1 group

To determine the function of CCNG2 in the cell cycle, we initially detected the endogenous expression level of CCNG2 in HNSCC cells. We found that CCNG2 was down-regulated in Tu686 and FaDu cell lines compared with HuLa-PC cells (Fig. 4A). The intracellular CCNG2 mRNA and protein level was greatly increased by transfecting the CCNG2 specific plasmid (Fig. 4B, C). We also observed that overexpression of CCNG2 caused an increase in Tu686 cells at the G1 phase (from 60.9 to 68%) and a decrease in FaDu cells at G2/M phase (from 28.8 to 20.2%; Fig. 4D), which was consistent with the results of miR-17-5p inhibition (Fig. 2A). CCND1 and CDK6, which induce cell cycle progression in CCNG2-overpressed Tu686 cells, were reduced. CCNB1 was reduced in FaDu cells overexpressing CCNG2 (Fig. 4E). Taken together, these data suggest that CCNG2 inhibits cell cycle progress in HNSCC in vitro.

## Discussion

Cell cycle alteration is one of the factors that causes malignant cell behaviors observed in cancer, such as proliferation, invasion and chemo-resistance [4]. miR-17-5p is a member of the miR-17-92 cluster, which plays an important role in the tumorigenesis of different cancers. Kim et al. found that miR-17-5p regulates epithelial–mesenchymal transition (EMT) by targeting vimentin in colorectal cancer (CRC) [20]. In gastric cancer, miR-17-5p has been shown to promote cellular proliferation and invasiveness by targeting RUNX3 [21]. However, the cell cycle regulation mechanism mediated by miR-17-5p is still unclear, especially in HSNCC.

The expression of miR-17-5p was reportedly upregulated in the plasma and tissues of patients with gastric cancer [21]. Wang et al. reported that miR-17-5p is upregulated in laryngeal squamous cell carcinoma (LSCC) tissues and cell lines. More importantly, they found that compared with patients with lower miR-17-5p expression level, patients with higher miR-17-5p expression levels in LSCC tissues have a poorer survival rate [5]. Consistently, in the present study, we observed an increased level of miR-17-5p in HNSCC tissues and cell lines. Surprisingly, HNSCC patients with higher levels of miR-17-5p had a higher likelihood of recurrence compared with patients who had a lower level of miR-17-5p, suggesting that miR-17-5p is related to recurrence in HNSCC patients. There are also reports on the results with some conflicting conclusions. Kim et al. found that miRNA-17-5p expression was lower in primary CRC tissues with metastasis than in primary CRC tissues without metastasis [20]. Fan et al. identified miR-17-5p as a metastatic suppressor of basal-like breast cancer [22]. We speculate that the differences between the promotion and inhibition of miR-17-5p may attributed to the organ-specific function of miRNA [20] and different target genes from those regulated by miR-17-5p as described by Cloonan et al. [6].

In the present study, we found that miR-17-5p promoted cell cycle progression of HNSCC cells mainly by increasing the proportion of cells in G2/M phase and reducing the proportion of cells in S phase. More importantly, we verified miR-17-5p negatively regulated CCNG2 mRNA and protein expression by directly targeting its 3’UTR, indicating that miR-17-5p might act as a tumor promoter in HNSCC. However, other teams have reported different mechanisms by which miR-17-5p promotes cell cycle progression. Cloonan et al. found miR-17-5p acts specifically at the G1/S-phase cell cycle boundary by targeting more than 20 genes involved in the transition between these phases [6]. Li et al. found that miR-17-5p promoted cell proliferation by promoting cell cycle G1/S transition and inhibiting ovarian cancer cell apoptosis, while inhibition of miR-17-5p resulted in the opposite [18]. Zhu et al. found that inhibition of miR-17-5p in pancreatic cancer cells resulted in a higher proportion of cells within the G1 phase and less in the S phase, leading to impaired proliferation of the cancer cells [9]. The results of these studies demonstrate that miR-17-5p mainly acts on the G1/S boundary in promoting cell cycle progression. Although the mechanism of action in our study is different from that reported by other teams, we all elucidate the role of miR-17-5p in promoting cell cycle progression. This difference may be largely due to the role of miR-17-5p target gene in cell cycle regulation.

Although most of the evidence in the literature supports a role for CCNG2 in limiting G1/S phase transition [23, 24], there are indications that CCNG2 could participate in G2/M regulation [23, 25, 26]. We previously proved that CCNG2 siRNA application in LSCC cell lines contributes to the increased G2/M phase proportion [16]. This is consistent with our findings that the overexpression of CCNG2 in FaDu cells led to a decrease in the G2/M phase proportion.

There are several limitations to our study. There are many targets of miR-17-5p other than CCNG2. It is possible that multiple mechanisms are at play. Additional in vivo research should be conducted if miR-17-5p and CCNG2 are to be considered therapeutic targets in HNSCC treatment. Although the direct regulatory relationship between the miR-17-5p and CCNG2 was confirmed by the dual-luciferase reporter assay, cell rescue experiments and in vivo animal experiments are needed for further verification.

## Conclusion

Clearly, our results demonstrate that miR-17-5p regulates the cell cycle of HNSCC cells by directly targeting CCNG2 and is related to recurrence in HNSCC patients. Further exploration of these molecules and a better understanding of miR-17-5p will help develop more effective therapies against HNSCC.

## Data Availability

All the source data supporting the findings of this study are available from the corresponding author upon reasonable request.

## Abbreviations

HNSCC: Head and neck squamous cell carcinoma
Cyclin G2: CCNG2
RT-qPCR: Real-time quantitative polymerasechain reaction
miRNAs: microRNAs
UTR: Untranslated region
LSCC: Laryngeal squamous cell carcinoma
EMT: Epithelial–mesenchymal transition
CRC: Colorectal cancer
